# The incidence of Cushing’s disease: a nationwide Swedish study

**DOI:** 10.1007/s11102-019-00951-1

**Published:** 2019-02-25

**Authors:** Oskar Ragnarsson, Daniel S. Olsson, Dimitrios Chantzichristos, Eleni Papakokkinou, Per Dahlqvist, Elin Segerstedt, Tommy Olsson, Maria Petersson, Katarina Berinder, Sophie Bensing, Charlotte Höybye, Britt Edén Engström, Pia Burman, Lorenza Bonelli, Cecilia Follin, David Petranek, Eva Marie Erfurth, Jeanette Wahlberg, Bertil Ekman, Anna-Karin Åkerman, Erik Schwarcz, Ing-Liss Bryngelsson, Gudmundur Johannsson

**Affiliations:** 1 Department of Internal Medicine and Clinical Nutrition, Institute of Medicine at Sahlgrenska Academy, University of Gothenburg, and The Department of Endocrinology, Sahlgrenska University Hospital, Gröna Stråket 8, 413 45 Gothenburg, Sweden; 2 Department of Medicine, Umeå University, 901 87 Umeå, Sweden; 3 Patient Area Endocrinology and Nephrology, Inflammation and Infection Theme, Karolinska University Hospital, 171 76 Solna, Sweden; 4 Department of Molecular Medicine and Surgery, Karolinska Institutet, 171 77 Stockholm, Sweden; 5 Department of Medical Sciences, Endocrinology, Diabetes and Metabolism, Uppsala University Hospital, 751 85 Uppsala, Sweden; 6 Department of Endocrinology, Skåne University Hospital, 214 28 Malmö, Sweden; 7 University of Lund, 223 50 Lund, Sweden; 8 Department of Endocrinology, Skåne University Hospital, 222 42 Lund, Sweden; 9 Department of Endocrinology, Department of Medical and Health Sciences, Linköping University, 581 83 Linköping, Sweden; 10 Department of Internal Medicine, School of Medical Sciences, Örebro University, 702 81 Örebro, Sweden; 11 Department of Occupational and Environmental Medicine, Örebro University Hospital, 702 81 Örebro, Sweden

**Keywords:** Cushing’s syndrome, Epidemiology, Incidence, Validation

## Abstract

**Background:**

Studies on the incidence of Cushing’s disease (CD) are few and usually limited by a small number of patients. The aim of this study was to assess the annual incidence in a nationwide cohort of patients with presumed CD in Sweden.

**Methods:**

Patients registered with a diagnostic code for Cushing’s syndrome (CS) or CD, between 1987 and 2013 were identified in the Swedish National Patient Registry. The CD diagnosis was validated by reviewing clinical, biochemical, imaging, and histopathological data.

**Results:**

Of 1317 patients identified, 534 (41%) had confirmed CD. One-hundred-and-fifty-six (12%) patients had other forms of CS, 41 (3%) had probable but unconfirmed CD, and 334 (25%) had diagnoses unrelated to CS. The mean (95% confidence interval) annual incidence between 1987 and 2013 of confirmed CD was 1.6 (1.4–1.8) cases per million. 1987–1995, 1996–2004, and 2005–2013, the mean annual incidence was 1.5 (1.1–1.8), 1.4 (1.0–1.7) and 2.0 (1.7–2.3) cases per million, respectively. During the last time period the incidence was higher than during the first and second time periods (*P* < 0.05).

**Conclusion:**

The incidence of CD in Sweden (1.6 cases per million) is in agreement with most previous reports. A higher incidence between 2005 and 2013 compared to 1987–2004 was noticed. Whether this reflects a truly increased incidence of the disease, or simply an increased awareness, earlier recognition, and earlier diagnosis can, however, not be answered. This study also illustrates the importance of validation of the diagnosis of CD in epidemiological research.

**Electronic supplementary material:**

The online version of this article (10.1007/s11102-019-00951-1) contains supplementary material, which is available to authorized users.

## Introduction

Cushing’s disease (CD) is a rare disorder with an estimated annual incidence between 1.2 and 2.4 cases per million [1–7]. Studies on the incidence of CD are, however, few and usually limited by the small number of patients included [1–4, 6, 7]. Similarly, epidemiological studies on comorbidity and mortality are often based on small patient cohorts and/or short follow-up time [1, 2, 4, 5, 8–13]. The largest epidemiological study on mortality in patients with CD published so far included 320 patients in remission. Patients from four countries were included and the median follow-up time was 11.8 years [14]. In a recent meta-analysis, data from eight studies were pooled, resulting in a total of 700 patients [15]. Thus, knowledge on epidemiology and outcome in Cushing’s syndrome (CS), including CD, is still limited.

Since 1987, more than 99% of all diagnostic codes at hospital discharge are registered in the Swedish National Patient Register (Patient Register) [16]. The register holds a high quality with a positive predictive value (PPV) above 95% for diagnoses such as myocardial infarction [17], rheumatoid arthritis [18], and hip fracture [19]. Whether this also applies for CD is unknown.

Thus, the aim of the present study was to validate the diagnosis in patients with presumed CD in Sweden and to calculate the annual incidence of the disease.

## Methods

This was a retrospective study where the medical records of patients with presumed CD identified in the Swedish Patient Register were reviewed to validate the diagnosis and to estimate the incidence of CD in Sweden between 1987 and 2013.

### Identification of patients

During all hospital visits in Sweden, a diagnostic code for the main reason for the visit, as well as for all secondary diagnoses, are registered according to the International Classification of Diseases (ICD) system. In our study we used the following diagnostic codes to identify patients with CD in the Patient Register:

A. ICD-9 (between 1987 and 1996):
  - CS (255A) and/or
  - CS (255A) + benign neoplasm of pituitary gland and craniopharyngeal duct (227D).
B. ICD-10 (between 1997 and 2013):
  (c) CS (255A) and/or
  (d) CD (E24.0) + benign pituitary adenoma (D35.2) and/or
  (e) CS (E24.9) + benign pituitary adenoma (D35.2)

We recorded all occasions when one of these codes had been registered in connection with a hospital visit together with the unique social security number of the patient, date of the visit, and the name of the health care unit.

### Validation of the CD diagnosis

A list of the patients identified was sent to the eight university hospitals in Sweden where the patients had received their diagnosis and/or had been treated. The medical records were reviewed, and the diagnosis validated through collection of clinical, biochemical, imaging, and histopathological data by either an endocrinologist experienced in diagnosing and treating CS or a resident in endocrinology under supervision of an experienced endocrinologist. A standardized case reporting form was used at all the clinics to capture information on date of diagnosis, treatment, remission status, presence and treatment of hypopituitarism, hypertension, diabetes mellitus, and osteoporosis (Online Appendix Fig. S1). When a diagnosis of CD was not confirmed, the correct diagnosis was provided.

The diagnosis of CD was based on collection of following information: (a) clinical features where symptoms and signs such as central obesity, hypertension, muscle weakness, fatigue, and menstrual irregularities/amenorrhea were considered to be typical for CD; (b) biochemical analyses including measurements of urinary free cortisol, plasma ACTH, midnight serum/salivary cortisol, dexamethasone suppressions tests, corticotropin-releasing hormone test, and metyrapone stimulation test; (c) visible pituitary tumor on magnetic resonance imaging; (d) results from inferior petrosal sinus sampling; and (e) histopathological diagnosis.

Thereafter, the patients were assigned to one of the following five groups: (a) confirmed CD; (b) other forms of CS (e.g. cortisol-producing adrenal adenoma, ectopic ACTH-producing tumors, cortisol-producing adrenocortical cancer); (c) CD diagnosis possible but not confirmed; (d) diagnoses related to CS (e.g., suspected CS that was ruled out after evaluation, drug-induced CS); and (e) diagnoses unrelated to CS.

### Ethics

The study was approved by the Regional Ethical Review Board in Gothenburg, Sweden (reference number 145/11; approved 25 April 2013) and by the National Board of Health and Welfare, Sweden.

### Statistical analysis

Categorical variables are presented as number (*n*) and percentages, and continuous variables as mean ± SD or median [interquartile range (IQR); range]. For comparison of continuous variables between two groups, the unpaired t-test was used for normally distributed data and Mann–Whitney U-test for non-normally distributed data. For proportions, Pearson Chi square or Fisher’s exact test were used. A two-sided *P* value of < 0.05 was considered statistically significant.

The annual incidence of CD was calculated by dividing the number of patients diagnosed with CD by the mean number of inhabitants in Sweden during each specific year and is presented as the number of cases per million together with 95% confidence intervals (CIs). The mean annual incidence during the whole period (1987–2013) was calculated as well as the mean annual incidence during three 9-year periods (1987–1995, 1996–2004, and 2005–2013). Information on the number of individuals living in Sweden during the study period was collected from Statistics Sweden (http://www.scb.se/en).

Statistical analyses were performed with SPSS version 25.0 for Windows.

## Results

### Validation of the diagnosis

In total, 1317 patients had received a diagnostic code for CS between 1987 and 2013 in the Patient Register in Sweden. Review of patient records verified the diagnosis of CD in 534 patients (41%) whereas 610 (46%) did not have CD (Table 1). In 41 patients (3%) the diagnosis of CD was probable but could not be confirmed. Medical records for 132 (10%) patients were missing. The majority of study patients with missing data (112; 85%) had received the diagnostic code for CS before 1997 (ICD-9).

**Table 1:** Patients who had received a diagnostic code for (a) CS (255A), (b) CS (255A) + benign neoplasm of pituitary gland and craniopharyngeal duct (227D), (c) CD (E24.0), (d) CD (E24.0) + benign pituitary adenoma (D35.2), and/or (e) CS (E24.9) + benign pituitary adenoma (D35.2) in Sweden between 1987 and 2013

	No. of patients (%) (*N* = 1317)
CD	534 (41)
CD probable but not confirmed	41 (3)
Other causes of CS	156 (12)
Cortisol-producing adrenal adenoma	86 (7)
Ectopic ACTH-producing tumors	52 (4)
Cortisol-producing adrenal carcinoma	17 (1)
Primary pigmented nodular adrenocortical disease	1 (< 1)
Diagnoses related to CS	120 (9)
Suspected CS, ruled out after evaluation	86 (7)
Drug-induced CS	32 (2)
Glucocorticoid receptor resistance	2 (< 1)
Diagnoses, not related to CS	334 (25)
Non-functioning adrenal adenoma	44 (3)
Diabetes mellitus	38 (3)
Pituitary disease, not adenoma	24 (2)
Addison’s disease	14 (1)
Craniopharyngioma	12 (1)
Non-functioning pituitary adenoma	12 (1)
Congenital adrenal hyperplasia	9 (1)
Acromegaly	10 (1)
Prolactinoma	8 (1)
Neuroendocrine tumor (carcinoid)	8 (1)
Aldosterone-producing adrenal adenoma	7 (1)
Other endocrine diagnoses	26 (2)
Other unrelated diagnoses	122 (9)
Medical records missing	132 (10)

*ACTH* adrenocorticotropic hormone, *CD* Cushing’s disease, *CS* Cushing’s syndrome

Of the 534 patients with confirmed CD, typical signs and symptoms were documented in the medical records of 469 (88%) patients, 460 (86%) had documented biochemical analyses supporting the diagnosis, 287 (54%) had a visible pituitary adenoma on imaging, 176 (33%) had performed an inferior petrosal sinus sampling, and a histopathological diagnosis was available in 215 patients (40%).

Of 610 patients without CD, 156 (12% of the whole cohort) had endogenous CS of other causes, 86 (7%) had a suspected CS that was eventually ruled out after clinical and biochemical evaluation, and 32 (2%) had a drug-induced (iatrogenic) CS (Table 1). Three-hundred-and-thirty-four patients had diagnoses unrelated to CS (25% of the whole cohort).

### Alternative search strategy

The median (IQR; range) number of occasions that patients with confirmed CD had received a diagnostic code was 11 (6–18; 1–97) compared to 1 (1–2; 1–20) in patients with diagnoses unrelated to CS (*P* < 0.001). Eight-hundred-and-eight patients had received at least one of the predefined diagnostic codes on more than one occasion: 507 (63%) patients with CD and 235 (29%) without CD (Table 2). Of the total cohort of 1317 patients, 1022 (78%) had received the diagnosis as the main reason for their visit: 497 (49%) patients with CD and 419 (41%) without CD. By including only patients with a diagnostic code for CD on more than one occasion and at least once as a main reason for their visit, 709 patients would have been identified: 479 (68%) with CD and 181 (26%) without CD.

**Table 2:** Number of patients who had received a diagnostic code on more than one occasion and/or as a code registered as a main reason of the visit

No. of patients (%)								
	Total cohort (*N* = 1317)	Patients with ICD-9 and/or 10 code registered on > 1 occasion (*n* = 808)	Patients with ICD-9 and/or 10 code registered as a main reason for the visit (*n* = 1022)	Patients with ICD-9 and/or 10 code registered on > 1 occasion and ≥ 1 time as a main reason for the visit (*n* = 709)	Patients with ICD-10 code registered (*n* = 731)	Patients with ICD-10 code registered on > 1 occasion (*n* = 511)	Patients with ICD-10 code registered as a main reason for the visit (*n* = 653)	Patients with ICD-10 code registered on > 1 occasion and ≥ 1 time as a main reason for the visit (*n* = 485)
CD	534 (41)	507 (63)	497 (49)	479 (68)	423 (58)	397 (78)	412 (63)	390 (80)
Other forms of CS	156 (12)	99 (12)	119 (12)	78 (11)	80 (11)	44 (9)	66 (10)	36 (7)
Diagnoses related to CS	120 (9)	31 (4)	80 (8)	29 (4)	48 (7)	16 (3)	41 (6)	15 (3)
Diagnoses unrelated to CS	334 (25)	105 (13)	220 (22)	74 (10)	147 (20)	39 (8)	110 (17)	31 (6)
Files missing	132 (10)	44 (5)	79 (8)	32 (5)	22 (3)	7 (1)	15 (2)	6 (1)
CD possible but not confirmed	41 (3)	22 (3)	27 (3)	17 (2)	11 (2)	8 (2)	9 (1)	7 (1)

*CD* Cushing’s disease, *CS* Cushing’s syndrome, *PPV* positive predictive value

Of the 731 patients who had received an ICD-10 code for CD, 511 (70%) had been registered on at least two occasions: 397 (54%) with CD and 99 (25%) without CD (Table 2). By including only patients who had received an ICD-10 diagnostic code for CS on more than one occasion and at least once as the main reason for their visit, 485 patients would have been identified: 390 (80%) patients with CD and 82 (17%) without CD.

### Patients with confirmed CD

**Fig. 1:**
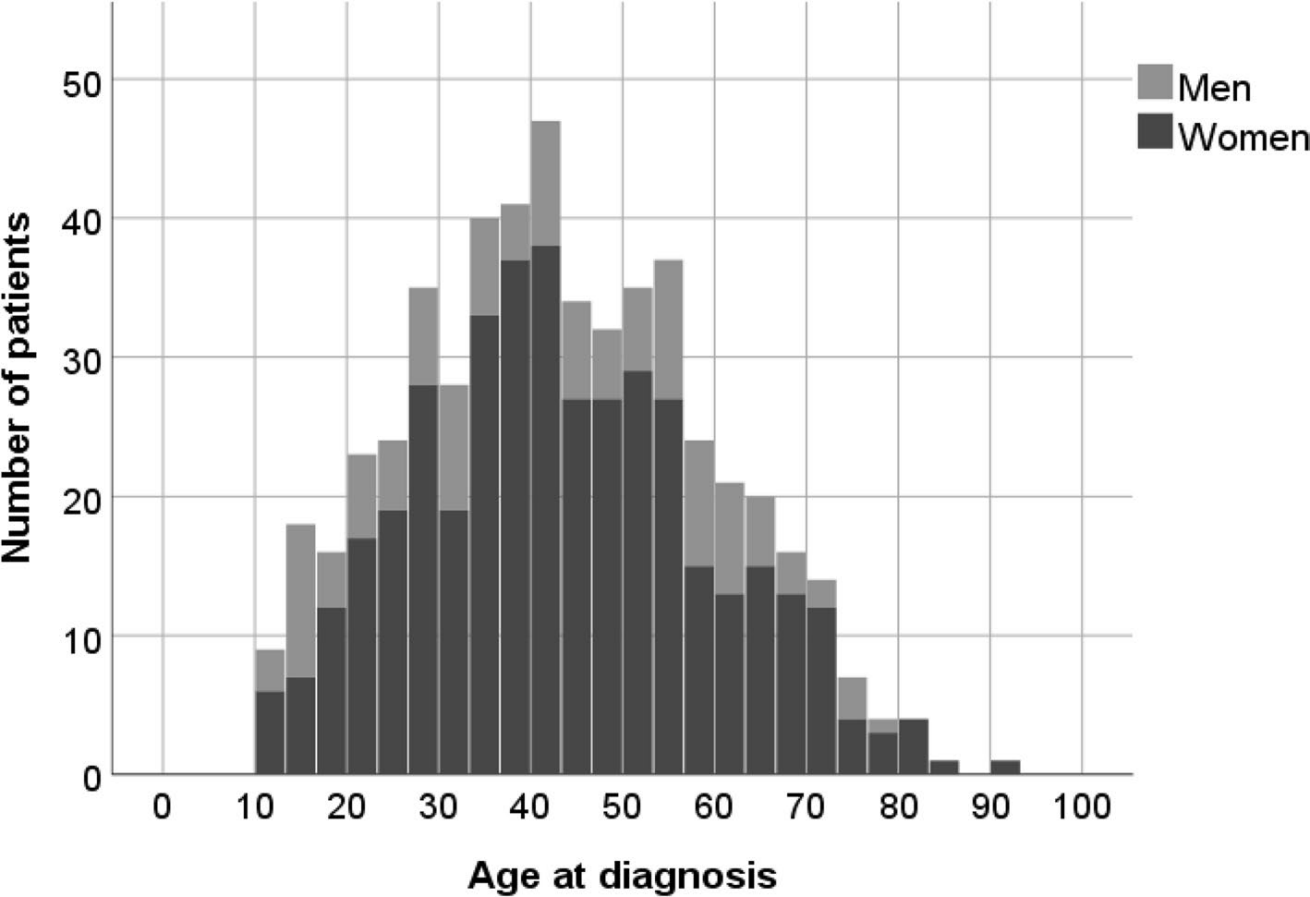
Age distribution at first diagnosis in patients with confirmed CD in Sweden

Of 534 patients with confirmed CD, 410 (77%) were women and 124 were men (23%). The mean (± SD) age at diagnosis was 43 ± 16 years (Fig. 1). Thirty-two (6%) patients were younger than 18 years at the time of diagnosis: 17 (53%) girls and 15 (47%) boys. Median (IQR; range) follow-up time was 10 (4–21; 0–55) years.

Information on treatment was available in 494 (93%) of patients with confirmed CD. The primary treatment modality was pituitary surgery in 381 (77%) patients, radiation therapy in 57 (12%) and bilateral adrenalectomy in 56 (11%). The primary treatment in 331 of 342 (97%) patients, diagnosed with CD 1990 or later, was pituitary surgery.

### Annual incidence of CD

**Fig. 2:**
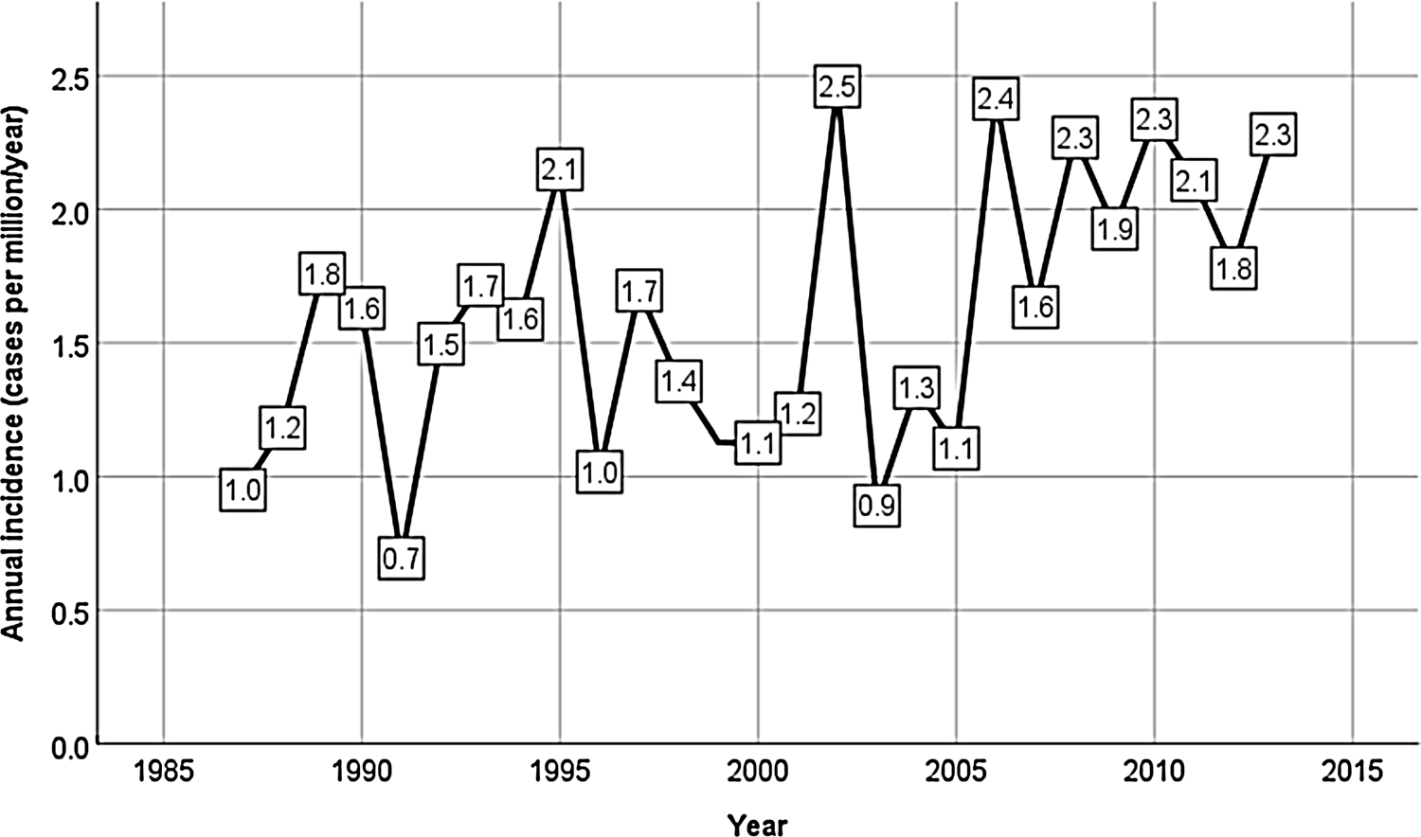
Annual incidence (cases per 1 million inhabitants per year) of CD in Sweden between 1987 and 2013

Of 534 patients with confirmed CD, 144 were excluded from the incidence analysis as their diagnosis was made before 1987. In total, 390 patients were diagnosed with CD in Sweden between 1987 and 2013. The mean (95% CI) annual incidence during the whole study period was 1.6 (1.4–1.8) cases per million (Fig. 2). The mean annual incidence was 1.5 (1.1–1.8) cases per million between 1987 and 1995, 1.4 (1.0–1.7) cases per million between 1996 and 2004, and 2.0 (1.7–2.3) cases per million between 2005 and 2013. The mean annual incidence was higher during the last time period than during the first (*P* = 0.022) and the second (*P* = 0.009) time periods.

## Discussion

This study illustrates the importance of validating diagnosis in patients with presumed CD from large health care registries such as national registries. Of 1317 patients identified in the Swedish Patient Register, approximately half had endogenous CS, of whom 534 had CD.

Although CD has been known as a clinical entity for more than 100 years, epidemiological studies evaluating incidence are surprisingly few and most often based on small numbers of patients (*n* = 19–73; Table 3) [1–4, 6, 7]. The largest study published to date on incidence was based on 188 patients diagnosed with CD between 1960 and 2005 in New Zealand [5]. The incidence in these studies varies between 1.2 and 1.8 cases per million/year [1, 3–6] with the exception of the study by Etxabe and Vazquez [2] who reported an incidence of 2.4 cases per million/year. Our result of 1.6 cases per million is therefore in agreement with most previous reports. In a recent study from the USA, an unexpectedly high incidence rate of 6.2–7.6 cases per million/year was reported [20]. That study was based on information from an insurance database where CD was defined as a CS diagnosis (as for ICD-9) plus the diagnosis of pituitary adenoma and/or hypophysectomy. There may be several reasons for this surprisingly high incidence, but most importantly, the medical records of the patients were not reviewed. It is therefore likely that the incidence was overestimated as indicated in our study.

**Table 3:** Summary of previous studies analyzing the annual incidence rate of CD

Author (year of publication) [reference]	Country	Period	No. of patients	Incidence (per million per year)	Comments
Etxabe and Vazquez (1994) [2]	Spain, Biscay province	1975–1992	41	2.4	
Lindholm et al. (2001) [1]	Denmark, nationwide	1985–1995	73 (99)	1.2 (1.7)	73 patients with confirmed CD, additionally 26 probably with CD
Raappana et al. (2010) [7]	Finland, northern	1992–2007	19	1.7	Medical records reviewed
Arnardottir and Sigurjonsdottir (2011) [3]	Iceland, nationwide	1955–2009	19	1.5	Medical records reviewed
Clayton et al. (2011) [4]	UK, Stoke-on-Trent	1958–2010	60	1.5	Medical records reviewed
Bolland et al. (2011) [5]	New Zealand, nationwide	1960–2005	188	1.3	Medical records reviewed at 4 main centers (> 90% of CS patients in the country)
Trjornstrand et al. (2014) [6]	Sweden, Västra Götaland County	2001–2011	25	1.8	Patients identified in the Swedish Pituitary Registry
Broder et al. (2015) [20]	USA	2009–2010	166	7.6 in 2009 6.2 in 2010	CD defined as the diagnosis of (ICD-9) CS + pituitary adenoma or hypophysectomy identified in an insurance database Medical records not reviewed Only patients ≤ 65 years No. of patients in the database used as denominator
Current study	Sweden, nationwide	1987–2013	534	1.6	390 patients included in the incidence analysis

*CD* Cushing’s disease, *CS* Cushing’s syndrome

We noticed a higher mean incidence between 2005 and 2013 compared to 1987–2004. Whether this reflects a truly increased incidence of the disease or simply an increased awareness, earlier recognition, and earlier diagnosis cannot be answered.

Between 1987 and 1996, discharge diagnoses in the Swedish National Register were coded according to the ICD-9 system, and thereafter according to ICD-10 criteria. In the ICD-9 criteria, only one code (255A) was available for CS. Thus, patients with all subtypes of the syndrome received the same diagnostic code irrespective of etiology. In the ICD-10 system, seven subtypes of CS were introduced including pituitary-dependent CD. Although this was an important improvement compared to the ICD-9 system, several CS diagnoses, especially cortisol-producing adrenal adenomas, did not receive a specific code. Since ICD-10 has a specific code for CD, we had expected that the reliability for the code would be much higher compared to ICD-9. However, just more than half of the patients with a diagnostic code for CD according to ICD 10 actually had the disease and 20% had a diagnosis completely unrelated to CS.

Combined search criteria have previously been proposed to identify patients for epidemiological research of rare diseases [21]. In fact, we have used this strategy successfully in epidemiologic studies on patients with Addison’s disease, craniopharyngioma, and non-functioning pituitary adenoma [22–24]. In the current study, we analyzed the influence of a combined search criteria on the accuracy of the diagnosis, i.e. by including only patients who had received the diagnosis more than once, only when the diagnosis was registered as a main cause of the visit, or a combination of both (Table 2). Unexpectedly, no combination resulted in acceptable accuracy for finding patients with CD: too many patients without CS were still identified and too many patients with true CS were not included. Similarly, by using a pituitary adenoma diagnosis in combination with CS (ICD-9) or CD (ICD-10), two-thirds of patients would have been missed in the ICD-9 system and almost half of the patients in the ICD-10 system.

The main strength of this study is the large number of patients where the diagnosis of CD was thoroughly evaluated. In fact, this is the largest study on incidence in patients with CD published so far. Another strength is the use of a national registry with almost 100% coverage of all patient visits in Sweden. There are, however, some limitations that need to be acknowledged. Medical records for 10% of the patients could not be retrieved despite vigorous efforts. Most of the files that were missing belonged to patients who had received the diagnosis more than 20 years previously. Also, the retrospective evaluation of the diagnosis of CD may have limitations. Although it can be considered a limitation that 41 patients had possible but unconfirmed CD, and were therefore not included in the incidence analysis, it can also be considered a strength that only patients with a confirmed diagnosis were included.

In conclusion, this nationwide study demonstrates the importance of validation of the diagnosis when using registries for epidemiological research in rare diseases such as CD. A correct diagnosis of CD was verified in less than half of the patients. Thus, the results from previous and future epidemiological studies using ICD coding for inclusion of patients with CD, as well as other rare and complex diseases, should be questioned.

## Electronic supplementary material

Below is the link to the electronic supplementary material.

Supplementary material 1 (DOCX 20 KB)
