# Supplementary material for: The incidence of Cushing’s disease: a nationwide Swedish study
Source: Pituitary. 2019 Feb 25;22(2):179–86. doi: 10.1007/s11102-019-00951-1 (PMC6418061; doi:10.1007/s11102-019-00951-1)
Supplement: Supplementary file 1 — Supplementary material 1 (DOCX 20 KB) [file 11102_2019_951_MOESM1_ESM.docx]

**APPENDIX**

**Validation of the diagnosis of Cushing’s disease is essential for epidemiological research**

Oskar Ragnarsson, Daniel S. Olsson, Dimitrios Chantzichristos, Eleni Papakokkinou, Per Dahlqvist, Elin Segerstedt, Tommy Olsson, Maria Petersson, Katarina Berinder, Sophie Bensing, Charlotte Höybye, Britt Edén Engström, Pia Burman, Lorenza Bonelli, Cecilia Follin, David Petranek, Eva Marie Erfurth, Jeanette Wahlberg, Bertil Ekman, Anna-Karin Åkerman, Erik Schwarcz, Gudmundur Johannsson

**Fig. S1.** Case report form.

**Cushing´s disease in Sweden - Epidemiology and comorbidities**

**Is the diagnosis of ACTH-producing pituitary adenoma (Cushing’s disease) confirmed^[[1]](#footnote-1)^?**

___ Yes ___ No If no, why? _____________________________

**The diagnosis of ACTH-producing pituitary adenoma was based on (choose one or more alternatives):**

___Clinical features ___Biochemical analyses ___Visible adenoma on MRI ___IPSS ___ PAD

**When was the patient diagnosed with ACTH-producing pituitary adenoma (year and month)?**____________

**What treatment has the patient received (if more than one operation/radiotherapy, provide date for all)?**

Pituitary surgery? ___ Yes ___ No When (year, mo) _________

Pituitary radiotherapy? ___ Yes ___ No When (year, mo) _________

Bilateral adrenalectomy? ___ Yes ___ No When (year, mo) _________

___________________________________________________________________________________________

**Information from the last clinical visit**

Date of the last clinical visit ______________ Name of clinic_________ _______________

**Is the patient cured/in remission?** ___ Yes ___ No

If yes, what is the evaluation based on (choose one or more alternatives):

___ a) Resolution of clinical features

___ b) Normalization of urinary free cortisol

___ c) Normalization of cortisol diurnal variation

___ d) Adequate cortisol suppression after dexamethasone suppression test

___ e) Adrenal insufficiency

___ f) Bilateral adrenalectomy

**Does the patient have medical treatment for Cushing’s disease?**

___ Yes ___ No If yes, which? _____________________________

**Does the patient have replacement therapy due to pituitary insufficiency?**

Thyroid hormone ___ Yes ___ No

Oestrogen/Testosterone ___ Yes ___ No

Growth hormone ___ Yes ___ No

Desmopressin ___ Yes ___ No

Glucocorticoids ___ Yes ___ No If yes, which type and dose________________

**Does the patient have medical treatment for hypertension?** ___ Yes ___ No

**Does the patient have medical treatment for diabetes mellitus?** ___ Yes ___ No

**Does the patient have medical treatment for osteoporosis?** ___ Yes ___ No

If yes, ___Calcium and vitamin D ___Bisphosphonates ___Another, what________________________

1. By review of medical records and evaluation of clinical, biochemical, imaging and histopathological data in connection with the diagnosis of Cushing’s disease. [↑](#footnote-ref-1)
